# Neighbourhood-built environment and cognitive or social health in older adults with mild cognitive impairment or dementia: an umbrella review

**DOI:** 10.1186/s12877-025-06693-z

**Published:** 2025-11-15

**Authors:** Janissa Altona, Henrik Wiegelmann, Emily Mena, Benjamin Schüz, Karin Wolf-Ostermann

**Affiliations:** 1 Institute for Public Health and Nursing Research (IPP), Bremen, Germany; 2 Health Sciences Bremen, Bremen, Germany

**Keywords:** Dementia, Alzheimer’s disease, Mild cognitive impairment, Neighbourhood-built environment, Cognitive and social health

## Abstract

**Background:**

Recent studies underscore the importance of the neighbourhood-built environment (NBE) for the cognitive and social health of older people with mild cognitive impairment (MCI) or people living with dementia (PlwD). While previous overview reviews have provided valuable insights, they often focus narrowly on either objective environmental features or subjective experiences and typically lack an integrated perspective. This umbrella review addresses this gap by systematically examining how specific NBE aspects (a) influence the cognitive and social health of older people with MCI or PlwD, and (b) subjective experiences by PlwD and their caregivers. By combining these perspectives, the review aims to support the development of dementia-friendly neighbourhood design and planning.

**Methods:**

To answer these questions, an umbrella review was performed. Scopus, MEDLINE (Pubmed), APA PsychINFO (Ebesco), CINAHL Complete (Ebesco), Cochrane Library, and Epistemonikos databases were used for the systematic literature research. We included peer-reviewed reviews or meta-analyses (quantitative, qualitative, or mixed-method studies) in German or English.

**Results:**

Ten reviews with 364 primary studies were identified. Reviews predominantly included quantitative studies, but also qualitative studies. The primary focus of the reviews was on the positive and negative influences of the NBE on MCI and/or dementia. Subjective experiences on social health targets were also addressed, but received less attention.

**Conclusions:**

The results of the reviews, although heterogeneous, highlight potential relationships between various NBE aspects and the cognitive and social health of older people with MCI or PlwD. Clear associations were identified for certain NBE features—such as green spaces and transportation infrastructure—which demonstrate positive influences on both cognitive functioning and social participation. These findings emphasise the importance of considering both objective environmental characteristics and the subjective perceptions of PlwD and their caregivers when designing dementia-friendly neighbourhoods. By doing so, this umbrella review contributes evidence-based guidance to support autonomy and independent living for people with MCI or dementia. Further research is needed to explore the specific influence of individual NBE aspects on social health and the lived experiences of PlwD and their caregivers.

**Supplementary Information:**

The online version contains supplementary material available at 10.1186/s12877-025-06693-z.

## Background

Dementia is an age-related neurodegenerative disease and in light of demographic aging processes, it poses a significant challenge for health care, nursing care, and social systems [1–3]. In Germany, approximately 1.8 million people are living with dementia (PlwD), and this figure could rise to 2.8 million by 2050. This is equivalent to an annual increase of 25,000 to 40,000 per year, or 70 to 110 PlwD per day. Two-thirds of the people diagnosed with dementia in Germany live in private residences [4], and to a large extent, close relatives (mostly family members, predominantly female) take over the care work. Of these, only 50–60% are without further formal support such as home care services [5]. Barriers and opportunities in the neighbourhood-built environment (NBE) can play a crucial role in the well-being, quality of life, self-determination, and social participation of older adults with mild cognitive impairment (MCI) or PlwD [6]. In this umbrella review, the term NBE refers specifically to the human-made physical features and infrastructure within a local, walkable area surrounding a person’s residence, typically at the scale of a neighbourhood. This includes elements such as residential and commercial buildings, local streets and intersections, sidewalks, parks, squares, street lighting, signage, and street furniture. By focusing on the neighbourhood scale rather than the broader city or town level, the aim is to better identify and understand the built environment factors most directly affecting daily lives and behaviours of older adults with MCI or PlwD.

Numerous studies demonstrate that older individuals residing in environments that promote social interactions, physical activities, and cognitive stimulation experience positive effects on their cognitive health [7–9]; while physical health is also influenced by these environmental factors, this review specifically focuses on cognitive and social health because these domains are most directly affected by dementia and most relevant for dementia-friendly design. Cognitive health here refers to the general cognitive functioning of older adults with MCI or PlwD. Even though cognitive health may be limited in people with MCI or dementia, it can still be supported to maintain daily functioning. Cognitive aging circumscribes the normal process of cognitive decline in aging without any reason for brain diseases [10]. In contrast, social health is a broader concept reflecting human capacities to engage in social life, including dignity, mutuality, and resilience [11]. Huber et al. 2011 distinguish three dimensions of social health in dementia: (1) capacity to fulfill one’s potential and obligations, (2) managing life despite the disease, and (3) participation in social activities [12, 13]. Importantly, there is a reciprocal relationship between cognitive and social health: cognitive health supports social engagement, while social health can positively influence cognitive functioning [14, 15].

According to Mitchell et al. (2010), PlwD are at risk of becoming housebound if they cannot safely use their immediate environment, highlighting the direct link between NBE features and autonomy. They also point to the positive influence of the outdoor environment on the physical and mental health, self-determination, well-being, and cognitive functioning of PlwD. In this sense, they describe dementia-friendly neighbourhoods as safe, comfortable, and engaging spaces that support everyday life for PlwD and others. Their qualitative study identified six principles for dementia-friendly design: familiarity, recognisability, distinctiveness, accessibility, comfort, and security [16].

Building on this, Wu et al. (2020) explored the perspectives of PlwD and their caregivers, identifying key components of dementia-friendly neighbourhoods—including care services, transportation, shops, integrated information, and opportunities for social participation. Similarly, Ward et al. (2017) concluded that neighbourhood characteristics can either support or hinder social engagement, thereby influencing social health and the risk of isolation for PlwD. Recent cohort and population-based studies further confirm that better access to recreational areas, walkability, and proximity to essential facilities are associated with a lower likelihood of progressing to severe dementia [6, 17–19].

The World Alzheimer’s Report 2020 underlines the role of the (built) environment, stating that “design for dementia is 30 years behind the physical disability movement - and that this must change” [20]. It highlights how dementia-friendly environments can enhance quality of life, self-determination, and alleviate psychological and behavioural symptoms, which also supports family caregivers and helps stabilise home care arrangements [20, 21]. There is therefore growing consensus that urban planning and architecture should integrate dementia-friendly principles to promote healthier living environments [22].

Despite this, existing research remains fragmented and lacks a comprehensive synthesis of how specific NBE features impact both cognitive and social health in PlwD. Moreover, few reviews explicitly combine objective environmental characteristics with the subjective experiences of PlwD and caregivers. This limits the translation of research into actionable design and policy recommendations. Therefore, our umbrella review aims to bridge these gaps by systematically examining (1) which NBE aspects influence the cognitive and social health of older people with MCI or PlwD, and (2) what subjective experiences are made by PlwD and/or caregivers with NBE features.

To achieve this, we focus on clearly defined and measurable NBE aspects—such as green and blue spaces (availability of natural elements, e.g., trees/lakes), street network connectivity, transport infrastructure, mixed land use (diverse destinations and facilities), and pedestrian and cycling infrastructure—as these can be identified relatively unambiguously across studies [23]. While our primary aim is to synthesise evidence across objective measures and subjective perceptions, we also draw on the conceptual framework proposed by Frank et al. (2019) [24], which links NBE factors to dementia outcomes through health-promoting mechanisms of action, particularly via behaviours and exposures that affect biological responses and chronic diseases. For example, a well-developed transport infrastructure or pedestrian environment can increase physical and social activity, which in turn may reduce systemic inflammation and stress, thereby lowering dementia risk [24, 25]. By structuring our synthesis in this way, we aim to better understand and integrate both quantitative and qualitative perspectives on dementia-friendly neighbourhood design.

In summary, there is considerable potential for preventative strategies that incorporate NBE elements to delay cognitive decline and support dementia care. By systematically aligning these two objectives, our review aims to bridge current research gaps and inform the development of dementia-friendly neighbourhood design and policy.

## Materials and methods

An umbrella review was conducted to summarise the evidence from published reviews. An umbrella review is a practical approach to getting an overview of a research field, primarily when multiple reviews are already published [26]. As the included reviews cover quantitative and qualitative primary studies this review type seems to be the most suitable methodological approach.

In order to summarise the results of previous reviews on the influence of specific NBE characteristics on older people with cognitive impairment, we set the following objectives based on the ‘simplified ecological model of cognitive health’ by Cerin (2019) [27], which shows the biological, behavioural, and environmental pathways of cognitive health and observes several internal and external factors. This model conceptualises cognitive health as shaped by complex interactions between external and internal factors, mediated by behavioural and biological pathways. External factors are grouped into three main domains: (1) the built and natural environment (e.g., density, street connectivity, mixed land use, transport infrastructure, pedestrian and cycling infrastructure, and natural green/blue spaces); (2) environment by-products such as air pollution, noise, and visual complexity; and (3) activity locations and mobility behaviour (e.g., walking, cycling, use of public transport). These external factors influence lifestyle behaviours—such as physical, mental, and social activities, sedentary behaviour, sleep, and diet—which then affect internal health pathways, including cardiometabolic health (blood pressure, adiposity, vascular events) and brain health (neurodegeneration, neuroplasticity, brain connectivity). To keep the scope of this umbrella review focused and manageable, we concentrated specifically on the external factors of the model—that is, characteristics of the built and natural environment and related by-products—as these are modifiable at the neighbourhood level and thus highly relevant for public health and urban planning aimed at supporting cognitive health in older adults. Internal factors (e.g., cardiometabolic health, brain health, and genetic predispositions) were considered as part of the explanatory pathways but were not the main focus of our synthesis.

We followed the Joanna Briggs Institute (JBI) Guidelines for umbrella reviews and the Preferred Reporting Items for Systematic Reviews and Meta-Analyses [28], and the Cerin (2019) ecological model of cognitive health was used to classify the data. According to the JBI, an umbrella review should provide an overall picture of results for a specific research question and can show a broader picture of insights, compared to a systematic review or meta-analysis. The review protocol including search terms has been published on PROSPERO (ID CRD42022299995).

**Table 1:** Characteristics of includes studies (*n*=10)

Author (year)	Review type	Main research aims	Studies and participants	Countries	Databases used (Range of years included)	Types of included studies	Neighbourhood built environment (NBE) categories	Quality appraisal
Besser, Lilah (2021)	Rapid Review	To identify evidence for benefits to cognition and brain structure/function due to green space exposure over life-course	22 quantitative studies In total, 979.501 participants (Range: 72–678.000.000)	USA, UK, China, Spain, Canada, Bulgaria, Germany, New Zeeland, Spain/UK/Netherlands	PubMed, Embase, Web of Science Core Collection (2012-2020)	all quantitative studies (mainly cross-sectional)	Natural green/blue space	Not conducted
Besser, Lilah et al. (2017)	Systematic Review	To review publications on the neighborhood social environment and BE and cognition in older adults (aged 45 years and older)	Six quantitative studies on BE In total, 13.237 participants (Range: 64-6.518.518)	USA, UK, the Netherlands, Japan, Singapore	PubMed, Web of Science (all databases), ProQuest Dissertation, Theses Global database (1989-2015)	all quantitative studies (mainly cross-sectional)	Density, Street network connectivity, Mixed land use, Pedestrian and cycling infrastructure, Transport infrastructure, Natural green/blue space, Neighbourhood physical disorder	ROBINS-I (Risk Of Bias In Non-randomised Studies - of Interventions)
Chen, Xi. et al. (2021)	Systematic Review	To provide a synthesis of the evidence on modifiable features of NBE associated with cognition and dementia risk in older adults	37 quantitative studies In total, 3.633.146 participants (Range: 64-2.165.165.268)	USA, UK, China, Japan, Australia, Mexico, Canada, Singapore, other European countries, multiple countries	MEDLINE Complete (Ebsco), Academic Search Ultimate (Ebsco), SPORTDiscus (Ebsco), Global Health (Ovid), Embase (Ovid), APA PsycINFO (Ebsco), and CINAHL Complete (Ebsco) (1989-2020)	all quantitative studies (mainly cross-sectional)	Density, Mixed land use, Transport infrastructure, Natural green/blue space, Neighbourhood physical disorder	Effective Public Health Practice Project Quality Assessment
De Keijzer, Carmen et al. (2016)	Systematic Review	To systematically evaluate observational evidence on the association between long-term exposure to green space (including parks, gardening, etc.) and cognition over the life span	13 quantitative studies In total, 14.128 participants (Range: 17-2.623.623)	USA, UK, Sweden, Spain, Australia	Medline, Scopus	all quantitative studies (cross-sectional and longitudinal)	Natural green/blue space	A non-validated quality appraisal tool used
Gan, Daniel R.Y. et al. (2021)	Scoping Review	To provide a synthesis of literature on dementia-friendly efforts as it relates to the neighborhood-built environment and its behavioural/psychosocial outcomes	29 studies; total participants: 34.979 (Range: 5–21.008.008)	UK, Canada, Sweden, Japan, the US, Hong Kong, Australia, New Zealand	AgeLine, APA, PsychINFO,Cumulative Indexto Nursing and AlliedHealth Literature,Global Health,Medical Literature Analysis,Retrieval System Online, Scopus	half of the studies werequalitative studies	Wayfinding/Getting lost/Losing orientation, Mixed land use (diverse destinations and facilities), Natural green/blue space	Not conducted
Keady, John et al. (2012)	Realist Review	To detail the essential findings and messages that link the experience of living with dementia to the neighborhood	18 studies (14 articles, two books, two reports); total participants: 199	UK	PsycARTICLES, CochraneDatabase systematicreviews, British Nursing Index, and Archives - 2010, Embase, Ovid Medline, Ovid Online, Social Care Online, Social Policy and Practice, Assia, Cinahl plus ebesco, Web of Science	qualitative, and quantitative, and mixed-method studies	Wayfinding/Getting lost/Losing orientation, Natural green/blue space	Not conducted
Kim, Jihye et al. (2021)	Scoping Review	To identify and provide information on the types of physical environments that can change the cognitive function of the elderly over 65.	12 studies; total participants 16.041 (Range: 63-7.000.000)	US, Canada, Brazil, China, France, Scotland, Singapore, Spain, Sweden, UK	CINAHL, Embase,PubMed, PsycINFO	quantitative studies	Density, Street network connectivity, Pedestrian and cycling infrastructure, Natural green/blue space	Not conducted
Mmako, Nkolika Janet et al. (2020)	Mixed studiesreview	To collate and review the evidence on ways people living with dementia in the community engage in and benefit from contact with green spaces and the various mechanisms by which these benefits arise	19 studies; total participants 618 (range: 5–88) Four quantitative studies (comparative cross-sectional, descriptive)	The UK, the Netherlands, Norway, Canada, Sweden, US	CINAHL, Medline,Web of Science, PsycINFO, Environment Complete, Psychology, Behavioral Sciences Collection, Sociology Source Ultimate, Academic Search Ultimate, Emerald Insight	qualitative, quantitative, and mixed-methods studies	Natural green/blue space	Mixed Methods Appraisal Tool (MMAT)
Sturge, Jodi et al. (2021)	Scoping Review	To summarize and identify gaps on what is known about how people with dementia who live at home perceive the social and built environment, outside the house, and how features of these environments contribute to their well-being	23 studies; total participants 620 (range: 6–67)	UK, Sweden	Scopus, PubMed,PsycINFO, Webof Science, GoogleScholar	qualitative studies	Wayfinding/Getting lost/Losing orientation, Natural green/blue space	Not conducted
Zhao, Yong-Li et al. (2021)	Systematic Review + Meta-analysis	To investigate the modifiable natural, physical and social environment factors from observational studies which are associated with dementia and cognitive impairment	185 quantitative studies The total number of participants as well as the range of sample sizes, was not accessible	n/a	PubMed, EMBASE, Web of Science, PsychINFO	all quantitative studies	Natural green/blue space, Density, Street network connectivity, Mixed land use	Newcastle Ottawa Scale (NOS)

### Search strategy

**Fig. 1:**
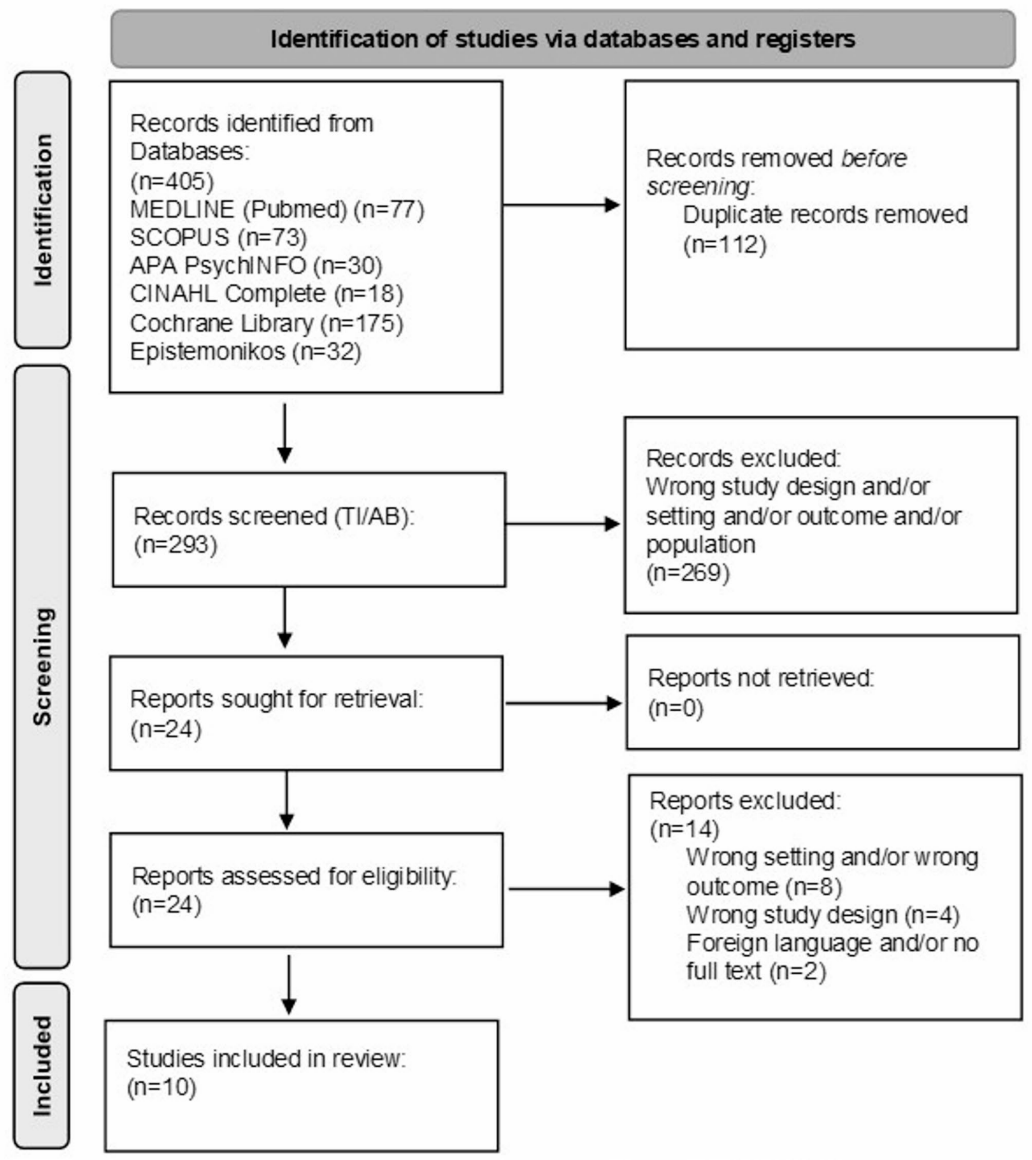
PRISMA 2020 flow diagram of the selection of publications for the review

The search strategy included terms and synonyms for (1) dementia (e.g., Alzheimer’s disease, mild cognitive impairment, cognitive aging); (2) built environment (e.g. neighbourhood design, physical environment). An example of the entire search string for MEDLINE (Pubmed) can be found in Appendix A. We limited our search to reviews written in English or German, without restriction on publication year. Only peer-reviewed reviews or meta-analyses of quantitative, qualitative, or mixed-method studies were included. We also searched Google Scholar and screened the first 250 results to ensure no relevant studies were missed. Exclusion criteria were all primary studies, comments, or editorials (Appendix A, Table 1). The literature search was conducted in December 2021 by two reviewers (JA, HW) using five databases most relevant to the scope of this review, including Scopus, MEDLINE (Pubmed), APA PsychINFO (Ebesco), CINAHL Complete (Ebesco), Cochrane Library and Epistemonikos. The following PRISMA flow chart shows the search selection process in detail (Fig. 1).

### Data extraction

We used a narrative synthesis to extract the data and followed the Joana Briggs Institute (JBI) guidelines [29]. An analysis was conducted to identify and categorise key themes and patterns across the included reviews. This involved multiple readings of the extracted data, coding of significant findings, and grouping them into themes. The identified themes were integrated to create a coherent narrative summarising the state of the evidence. This involved comparing and contrasting findings from different reviews, highlighting consistent findings, discrepancies, and gaps in the literature concerning the research question. This implies that quantitative and qualitative data did not undergo repeated analyses. Results were presented in a structured manner, with each topic discussed in detail. Relevant citations and data points from the included reviews were used to support the narrative, ensuring a comprehensive and transparent synthesis. First, the reviews were classified into an Excel dataset to get an overview of the review types, main research aims, studies and participants, countries, databases used, included studies, and the applicable NBE categories. Second, the main results of the reviews were extracted into a Word document and were categorised into the NBE categories by Cerin (2019). The results were additionally separated into qualitative and quantitative results. Two researchers (JA, HW) discussed which parts and paragraphs to extract to provide new insights and to answer one of the research questions. The data were double-checked to avoid mistakes.

## Results

### Descriptive results

We identified 405 literature reviews with 364 primary studies in the five databases and removed 112 publications due to duplication. The reviews were published between 2012 and 2021. We excluded 269 publications after screening the titles and abstracts and reviewing the full texts of the remaining 24 publications. From these, we excluded 14 publications because of incompatible settings and/or outcomes (*n* = 8), study designs (*n* = 4), or languages other than English/German and/or the non-existence of the full text (*n* = 2). Finally, we included 10 reviews in a narrative synthesis. To avoid an overestimation of the results of the final sample, we also checked for duplicates at the primary study level wherever possible. The reviews primarily included quantitative studies but also some qualitative studies. We included one rapid review [30], one mixed studies review [31], one realist review [32], one systematic review and meta-analysis [33], three systematic reviews [7, 34, 35], and three scoping reviews [36–38].

The primary studies included in the reviews were mostly cross-sectional and longitudinal studies, focusing on natural green and blue spaces [7, 30, 31, 33–37]. Most of the included reviews have applied some quality appraisal. Table 2 provides an overview of the NBE categories mentioned in the studies. The JBI checklist was used to check the quality of the reviews [29]. The tool contains 11 questions regarding the quality of the included research syntheses. Two reviewers (JA, HW) scored the JBI questions independently, subsequently compared their results, and agreed on a common appraisal. We calculated the overall percentage of appraisal criteria met across all included studies, and findings were not weighted based on study quality. Table 2 shows the summary of the quality appraisal.

**Table 2:** Quality appraisal for included reviews using the Joanna Briggs Institute (JBI) critical appraisal tool

Author (year)	1 Is the review question clearly and explicitly stated?	2 Were the inclusion criteria appropriate for the review question?	3 Was the search strategy appropriate?	4 Were the sources and resources used to search for studies adequate?	5 Were the criteria for appraising studies appropriate? *	6 Was critical appraisal conducted by two or more reviewers independently?	7 Were there methods to minimise errors in data extraction?	8 Were the methods used to combine studies appropriate?	9 Was the likelihood of publication bias assessed?	10 Were recommendations for policy and/or practice supported by the reported data?	11 Were the specific directives for new research appropriate?
Besser (2021)	Y	Y	Y	Y	Y	N	N	Y	N	N/A	Y
Besser et al. (2017)	Y	Y	Y	Y	Y	U	N	Y	N	N/A	Y
Chen et al. (2021)	Y	Y	Y	Y	N	Y	Y	Y	N	N/A	Y
De Keijzer et al. (2016)	Y	Y	Y	Y	N	Y	N	Y	N	N/A	Y
Gan et al. (2021)	Y	Y	Y	Y	N/A	N/A	N	Y	N	Y	Y
Keady et al. (2012)	N	Y	Y	Y	N/A	N/A	N	Y	N	N/A	Y
Kim et al. (2021)	Y	Y	Y	Y	N/A	N/A	N/A	Y	N	N/A	Y
Mmako et al. (2021)	Y	Y	Y	Y	Y	Y	N	Y	N	N/A	Y
Sturge et al. (2021)	Y	Y	Y	Y	N/A	N/A	Y	Y	N	Y	Y
Zhao et al. (2021)	Y	Y	Y	Y	Y	Y	Y	Y	Y **	N/A	Y
Overall percentage of criteria met (Y)	90%	100%	100%	100%	40%	40%	30%	100%	10%	20%	100%

*N* NO, *N*/*A *Not applicable, *U *Unclear, *Y* Yes

* Had to be a validated appraising tool

** Publication bias was examined and adjusted

### NBE and cognitive/social health

We incorporated Cerin’s (2019) ecological model of cognitive health to classify NBE categories and examine their associations with health indicators [27]. Additionally, we introduced two new categories: ‘neighbourhood physical disorder’ and ‘wayfinding infrastructure’. Cognitive and social health were measured very heterogeneously in the primary studies of the included reviews, which was also emphasised in the reviews as a challenge to comparability. In most cases, the Mini-Mental State Examination (MMSE) was used to measure cognitive health. No standardised instruments were used for social health; instead, mainly qualitative methods on social networks or social support were conducted.

### Natural green and blue spaces

Green and blue spaces include environments that are either naturally developed or artificially created, such as parks or forests (green spaces) or lakes and rivers (blue spaces) [1].

#### Objective associations between natural green and blue spaces and cognitive health

All included reviews reported findings regarding green and blue infrastructures [7, 30–38], and the results on the relationship between green and blue spaces and cognitive health can be described as consistent. Quantitative results predominantly showed positive associations between natural green/blue space and cognitive health, cognitive functioning/impairment, or dementia prevention. This applies to cross-sectional as well as longitudinal studies. Three reviews focused exclusively on the benefits of green space exposure on cognitive health, brain structure/function, and dementia prevention [30, 31, 35].

Several studies emphasised the positive effect of neighbourhood green spaces on cognitive health. For instance, Chen et al. (2021) showed that more green space, as well as the availability of parks during childhood, have positive effects on cognitive health in later life [34]. Gan et al. (2021) showed a positive association between the availability of natural environments within the neighbourhood and less cognitive decline [37]. Similarly, Kim et al. (2021) reported a positive correlation between the availability of parks in childhood and less cognitive decline at later ages [36]. In a meta-analysis, Zhao et al. (2021) pointed out positive associations between more residential greenness and better cognitive health [33]. Their analyses of cohort studies showed that more greenness resulted in a 4% decrease in the risk for cognitive impairment and dementia prevalence. However, Besser et al. (2017) reported that no association between increased neighbourhood park area and cognitive health in adults aged 45 years and older could be found [7].

#### Subjective experiences of PlwD and informal carers with natural green and blue spaces and social health

Qualitative findings indicated that access to natural areas (parks, walkways) can encourage PlwD to go outside, fostering social participation and providing opportunities for a sense of agency, self-worth, and independence [31]. Sturge et al. (2021) pointed out that changes in natural green/blue spaces might negatively affect PlwD by causing disorientation [38]. Keady et al. (2012) highlighted that housing impacts the well-being and quality of life for PlwD through its connection to nature and access to the outdoors, which offer sensory stimulation and social interaction opportunities [32]. Instead, Gan et al. (2021) noted that PlwD are less motivated to visit natural green and blue spaces if these include barriers [37].

In summary, both quantitative and qualitative data predominantly support the positive effects of natural green and blue spaces on cognitive and social health. However, some negative outcomes are reported, particularly concerning the motivation and orientation of PlwD in these spaces. Overall, the findings suggest a strong benefit of natural environments, though the design and accessibility of these spaces are crucial to maximising their positive impact.

### Street network connectivity

Street network connectivity describes the ease or difficulty of navigating and reaching destinations within a neighbourhood. Better street network connectivity is characterised by fewer turns needed to access streets, integrated streets, mixed land use (library accessibility, sports venues, and food store availability), and fewer cul-de-sacs and dead ends [39]. Four reviews [7, 33, 36, 37] reported that better street network connectivity is associated with improved cognitive or social health.

#### Objective associations between street network connectivity and cognitive health

Besser et al. (2017) found street network connectivity to be positively associated with slower cognitive decline [7]. The review also emphasised an association with slower cognitive decline when there are wider footpaths and connections between streets. Another review indicated that road connectivity, combined with multipurpose facilities and pedestrian density, positively impacts cognitive health, particularly in immediate and delayed memory recall, visuospatial, construction, and language skills [36]. Zhao et al. (2021) also showed that higher street integration, combined with other NBE factors, benefits cognitive impairment and dementia prevention [33].

#### Subjective experiences of PlwD and informal carers with street network connectivity and social health

Qualitative findings by Gan et al. (2021) suggested that better street network connectivity leads to more frequent short outdoor walks and improved daily routine task performance [37], which are positively associated with mental well-being due to a sense of agency and social connection. Overall, the quantitative and qualitative findings suggest that enhancing street network connectivity not only facilitates easier movement within neighbourhoods but also promotes cognitive resilience and social engagement, thereby supporting healthier communities.

### Mixed land use

Mixed land use describes the diversity and availability of local amenities [40]. Five reviews suggest [7, 32–34, 37] that mixed land use positively impacts cognitive or social health.

#### Objective associations between mixed land use and cognitive health

Quantitative studies by Besser et al. (2017) showed a positive association between higher land use mix and decreased odds of dementia [7]. Specifically, they found positive associations between neighbourhood availability of community centers or an increased number of blocks to community resources and a slower cognitive decline. Gan et al. (2021) also found positive associations between diverse land use, combined with the use of natural space, on cognitive health. Moreover, they showed a positive influence of accessibility to libraries and walkability in general on cognitive health [37].

Chen et al. (2021) reported positive associations of mixed land use with cognitive functioning, cognitive health, or slower cognitive decline in walkable neighbourhoods with accessible community resources [34]. In contrast, Zhao et al. (2021) found no benefits of better food store availability, library accessibility, and a land use mix for preventing cognitive impairment and dementia [33].

#### Subjective experiences of PlwD and informal carers with mixed land use and social health

Keady et al. (2012) and Gan et al. (2021) underlined the importance of connections to local shops and organisations in the neighbourhood of PlwD, as these facilitate social exchange and maintain independence [32, 37]. Overall, while mixed land use shows promise in enhancing cognitive and social health outcomes through improved access to amenities and community resources, further research is needed to better understand its nuanced effects across different populations and contexts.

### Pedestrian and cycling infrastructure

Pedestrian and cycling infrastructure includes all pathways and bike paths within a specific area, considering factors such as quality (e.g. surface condition) and connectivity. The results of four studies [7, 33, 36, 37] predominantly suggested that there is a positive relationship between better pedestrian and cycling infrastructure and better cognitive status.

#### Objective associations between pedestrian and cycling infrastructure and cognitive health

Besser et al. (2017) found no association between faster cognitive decline and insufficient pedestrian infrastructure [7]. Kim et al. (2021) showed that better pedestrian infrastructure and road connectivity are associated with better maintenance of cognitive status [36]. The review of Zhao et al. (2021) identified several factors, such as the availability of more playgrounds, sports venues, food stores, libraries, higher neighbourhood walkability, and more sidewalk coverage, as preventive measures against cognitive impairment and dementia [33]. Another review identified a greater distance to cafes or post offices or a ‘poor micro-scale environment’ according to the Residential Environmental Assessment Tool (REAT) as risk factors for cognitive impairment and dementia prevalence [8].

#### Subjective experiences of PlwD and informal carers with pedestrian and cycling infrastructure and social health

According to Gan et al. (2021), the ability to go out for short or routine tasks without accompaniment positively influences mental well-being due to a sense of agency and social connection [37]. Additionally, walking has essential benefits for dyadic relationships (PlwD and their relatives), social exchange, and self-connections, and promoting feelings of belonging when ensuring a safe space [41]. The qualitative and quantitative findings underscore the importance of well-designed pedestrian and cycling infrastructure not only for enhancing mobility and accessibility but also for promoting cognitive health and fostering social interactions among people with MCI and PlwD.

### Wayfinding infrastructure

Wayfinding infrastructure describes the available support to help individuals orient themselves in a specific area or city [42]. PlwD often struggle with orientation, and both familiar and unfamiliar environments can be challenging depending on the dementia stage [42, 43]. Three reviews examined this NBE domain (Gan et al. 2021, Keady et al. 2012, and Sturge et al. 2021). Most findings demonstrated a positive relationship between a good wayfinding infrastructure and cognitive or social health.

#### Objective associations between wayfinding infrastructure and cognitive health

Gan et al. (2021) noted that repaving pedestrianised shopping streets to differentiate them from typical streets and using text signage to locate public toilets are helpful for wayfinding [37].

#### Subjective experiences of PlwD and informal carers with wayfinding infrastructure and social health

Qualitative findings by Gan et al. (2021) indicated that anxiety or insecurity due to stressors such as noise or unexpected obstacles can reduce activity spaces and lead to social isolation [37]. Keady et al. (2012) highlighted that changes in the NBE, such as alterations in bus routes, can inhibit PlwD and lead to ‘out of place’ feelings [32]. Factors such as navigability, legibility, safety, and environmental attractiveness are crucial for PlwD to orient themselves in the neighbourhood. In general, the use of outdoor spaces is a fluid and changing experience for PlwD. Sturge et al. (2021) showed that PlwD often rely on traffic lights and pedestrian flows to feel safe in traffic situations [38]. They also prefer areas with limited traffic and clearly marked, timed, and controlled pedestrian crossings. Another important factor in the wayfinding of PlwD is that similar-looking houses or buildings make orientation difficult. In general, PlwD have more difficulties finding their way than older adults without dementia. Therefore, street signs and path directions are useful for wayfinding and maintaining independence and social connections. Overall, the availability of well-designed wayfinding infrastructure plays a crucial role in supporting cognitive health by reducing navigation challenges and enhancing confidence in familiar and unfamiliar environments alike. Furthermore, it contributes to social health by facilitating easier access to community spaces and maintaining a sense of independence and connection for PlwD and people with MCI.

### Transport infrastructure

Transport infrastructure encompasses all public transport options available in a certain area. Effective public transport infrastructure is indicated by the availability of transport stops in close proximity, frequent departure times, and good connections to further public transport [44]. Overall, the findings of three reviews [7, 34, 38] suggested that the proximity of transport infrastructure is positively associated with cognitive health.

#### Objective associations between transport infrastructure and cognitive health

Besser et al. (2017) showed that transport infrastructure is positively correlated with slower cognitive decline when public transport stops are available [7]. The review by Chen et al. (2021) described negative associations between living closer to major roads and cognitive performance in later life [34]. However, they found positive associations between slower cognitive decline and accessible public transport, as well as good street connectivity and better cognitive health. No associations were found for several other indicators of transport infrastructure, such as cycling infrastructure, traffic safety, road tidiness, and handicap access.

#### Subjective experiences of PlwD and informal carers with transport infrastructure and social health

Sturge et al. (2021) pointed out that using public transportation can positively impact the self-confidence and independence of PlwD and indirectly benefit their social health [38]. However, public transport can also be stressful or scary when crowded.

In total, while transport infrastructure plays a critical role in supporting cognitive health by providing accessible and stress-free mobility options, its impact on social health among PlwD and their carers can vary depending on the quality of the transportation experience.

### Density

Density refers to both population density and building density, primarily existing in urban areas [45]. Results regarding the domain density were found in four studies [7, 33, 34, 36].

#### Objective associations between density and cognitive health

Most studies showed negative associations between higher density and cognitive health or dementia prevalence. Besser and colleagues (2017) reported associations between density and cognitive health [7], and found in one study that worse cognitive health was related to a more natural environment within the neighbourhood area, interpreted as an indicator of lower population density. Two other studies in this review found no associations between population density and cognitive health. Chen et al. (2021) presented four studies showing positive cross-sectional associations between urbanity (higher density) and better cognitive health and various cognitive subdomains [34]. However, two studies found no association between cognitive function and higher density. Kim et al. (2021) defined density as residential density and showed four studies suggesting that limited living space increases the risk of developing dementia or MCI [36]. They also found no differences in the risk of developing dementia between older adults living in rural and urban regions. Limited activity space, particularly in economically unequal neighbourhoods, increases the risk of developing dementia. The review also showed that among people living in confined spaces, 13.9% developed Alzheimer’s Disease (AD). These proportions increased to 21% when the analysis was adjusted for age, sex, education, and race. A meta-analysis by Zhao et al. (2021) summarised 35 studies and found that living in rural areas (lower population density) was associated with a higher risk of dementia and cognitive impairment. However, the quality of the included studies was rated as low [33].

In summary, while some studies suggest a negative impact of higher density on cognitive health and dementia risk, others show mixed or even positive associations. Factors such as living space constraints and neighbourhood characteristics also play significant roles.

### Neighbourhood physical disorder

Neighbourhood physical disorder, reported in two reviews [7, 34], describes factors such as vandalism, neglected buildings, and vacant land, which can lead to insecurity and feelings of being lost [46, 47].

#### Objective associations with neighbourhood physical disorder/ neighbourhood aesthetics and cognitive health

The quantitative results mainly showed that neighbourhood physical disorder negatively influences cognitive performance. Besser et al. (2017) reported a positive correlation between the deterioration of public spaces and faster cognitive decline [7]. Chen et al. (2021) found positive associations between poorer cognitive performance and perceived neighbourhood physical disorder [34]. They also reported that living in well-maintained neighbourhoods was associated with slower cognitive decline and better cognitive performance in old age. In addition, living in an aesthetic and pleasant neighbourhood was positively associated with better cognitive performance in old age. The review found no associations between cognitive health and objective composite measures of neighbourhood physical disorder. All in all, the reviews show that enhancing neighbourhood aesthetics and maintenance potentially contributes to better cognitive health outcomes in aging populations, highlighting the importance of urban planning and community development in promoting cognitive well-being.

The results on the NBE and cognitive or social health show some inconsistent findings, which may be explained by the multiple mediating effects of the associations between NBE and cognitive/social health. As the ‘simplified ecological model of cognitive health’ by Cerin (2019) [27] shows, there are also internal factors, such as brain and cardiovascular health or genetic impacts, that influence cognitive health that need to be considered.

### Summary of evidence

Tables 3 and 4. summarise the results of the included reviews using traffic light logic, following the recommendations of the JBI (2014) [29]. In addition, one to three plus signs were added for better readability. Table 3 shows the synthesised qualitative results. The results highlighted in green (three plus signs) indicate positive effects of the individual NBE categories, and the results highlighted in yellow (two plus signs) indicate contradictory results. Results highlighted in red (one plus sign) indicate negative effects. The evidence presentation of the quantitative studies deviates slightly from the JBI recommendation, as the primary studies are not primarily intervention studies. In this case, the green color indicates that there are already significant results that show a positive association between the NBE category and cognitive health or dementia prevention. Yellow indicates that there are still no precise or predominantly contradictory results (with considerably mixed results) for an association between the NBE category and cognitive health or dementia prevention. Red indicates that there are no significant results (regardless of the directional connection).

**Table 3:**
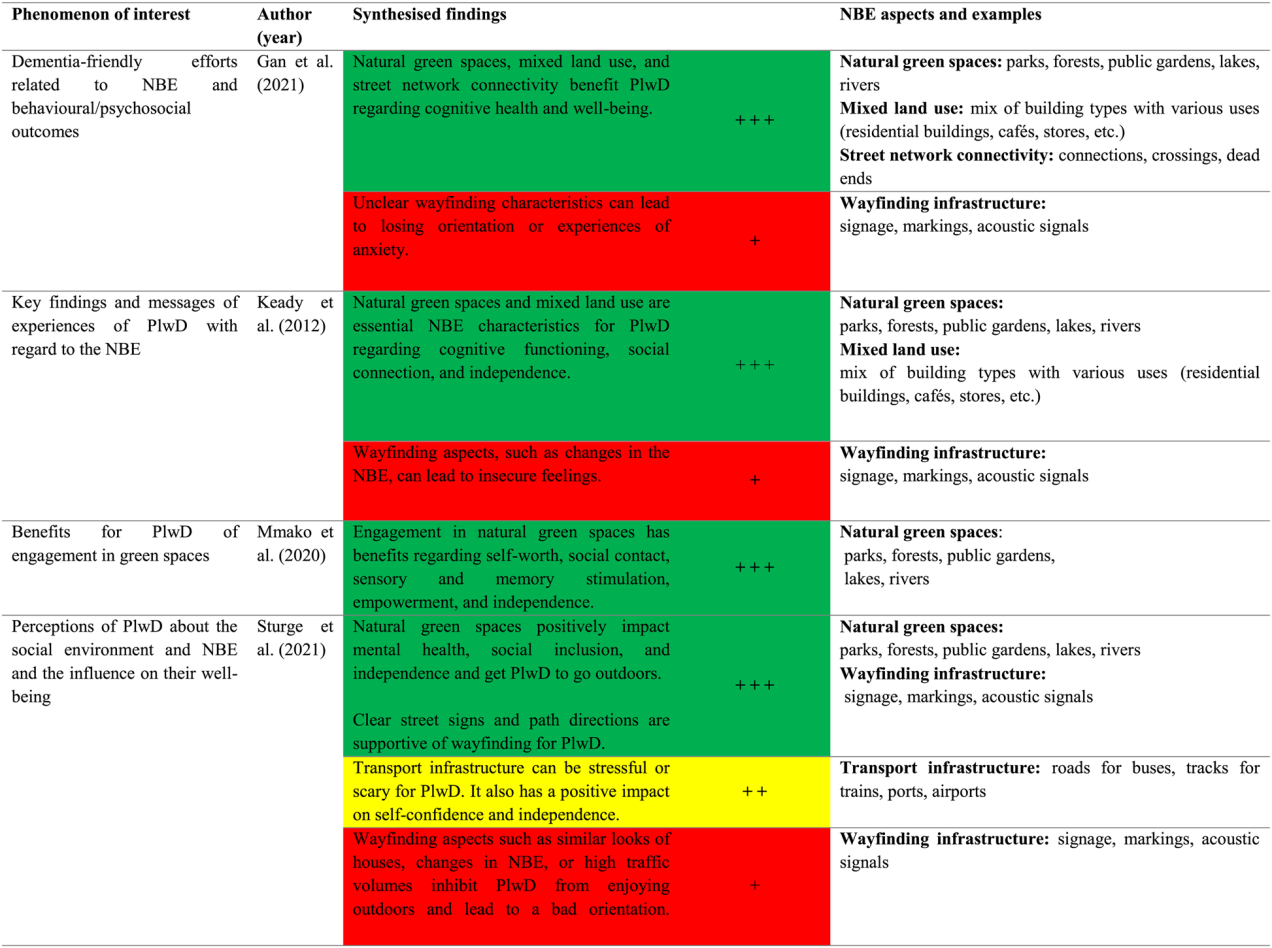
Summary of evidence from qualitative reviews

**Table 4.**
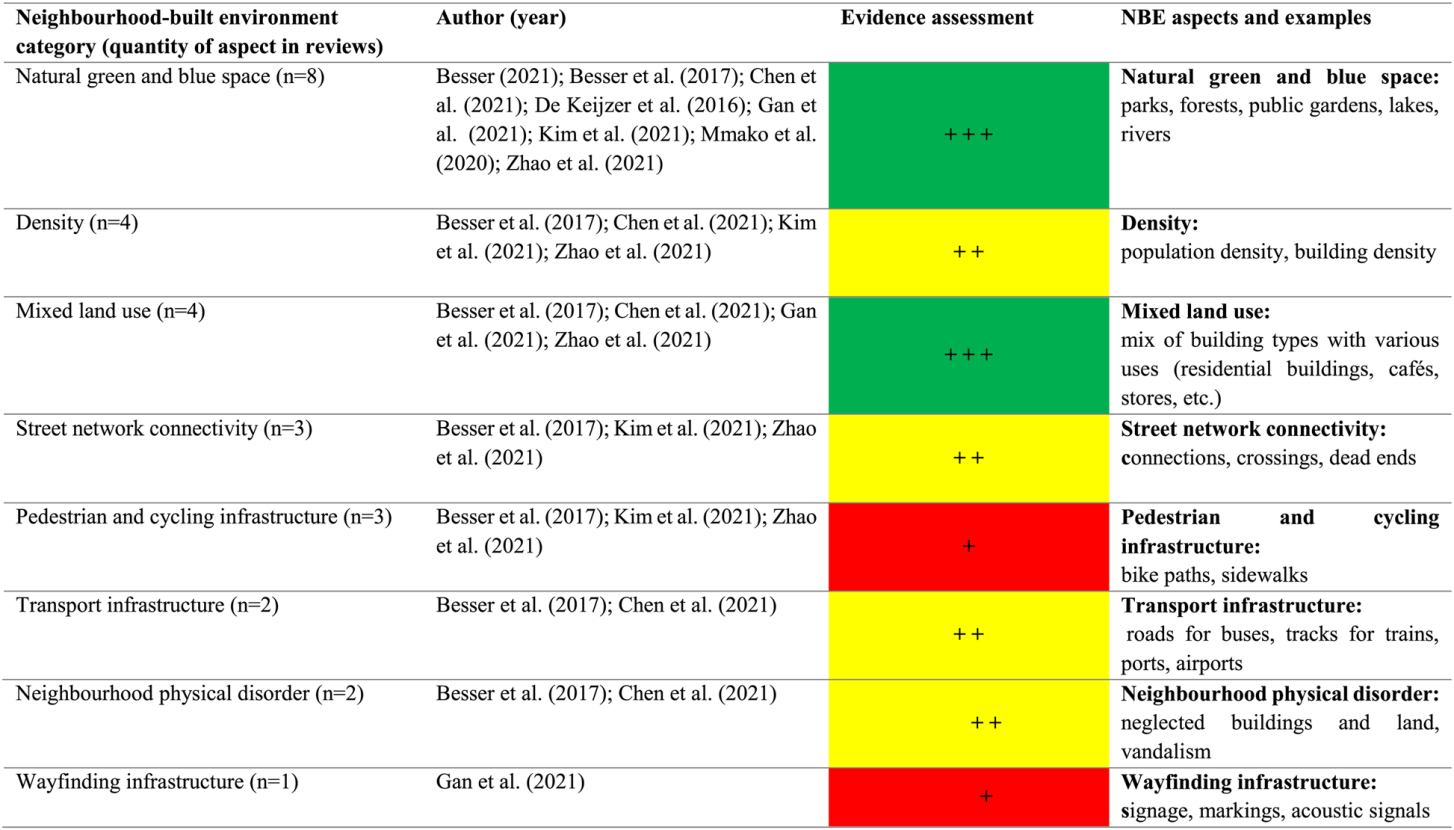
Summary of evidence from quantitative reviews

Qualitative (evidence) assessment based on indicators developed by the JBI [26]

It should be noted that the meaning of colours differs between qualitative and quantitative evidence, as the “red” category represents negative effects in qualitative syntheses but non-significant results in quantitative analyses. This distinction was intentionally maintained to ensure interpretative clarity within each evidence type while preserving overall visual consistency across tables.

An additional colour to display significant negative associations was not included, as these were very rare in the included reviews. According to the JBI guidance for umbrella reviews, the traffic light system is intended to provide a clear and simplified summary of the overall strength and consistency of evidence rather than to detail all possible directions of effect, which are better discussed narratively in the text. This approach avoids overemphasising findings based on limited evidence and keeps the visual summary balanced and transparent. The synthesised findings and evidence assessment column differ in their presentation due to the JBI standards. The qualitative evidence is listed by individual theses per author. The quantitative evidence is presented according to summarised findings and across authors.

Table 4 is not based on numerical values but on an overall qualitative assessment of the main conclusions and key findings.

## Discussion

This umbrella review synthesised qualitative and quantitative findings from 10 reviews and classified them into eight key neighbourhood-built environment (NBE) criteria. Adopting the six existing NBE categories of Cerin’s (2019) [27]‘simplified ecological model of cognitive health’, we categorised our findings and focused on the main external factors influencing cognitive health. By structuring our synthesis around this model, we could show how different NBE features interact with mediating behavioural and biological pathways, such as promoting physical activity or reducing stress, to support cognitive health.

Our synthesis also reveals several NBE aspects that are not considered in Cerin’s model, such as wayfinding infrastructure and neighbourhood physical disorder, which emerged inductively. These dimensions are under-researched but may importantly shape cognitive outcomes and warrant further investigation.

Overall, this review achieved two main objectives: first, to identify which NBE aspects influence the cognitive and social health of older people with MCI or PlwD; and second, to summarise how people with MCI, PlwD, and their caregivers experience these environments. The central takeaway from this synthesis is that research on NBE and cognitive health remains divided between objective environmental measurement and subjective experience. To bridge this gap, an integrated approach is required—one that connects measurable design attributes with lived experience to capture how people truly interact with their surroundings.

### Integrating objective and subjective perspectives

A key takeaway from this review is that understanding the NBE’s influence on cognitive and social health requires an integrated approach that combines objective environmental characteristics with subjective lived experiences. Previous reviews tended to treat these aspects separately, resulting in fragmented insights. Similarly, many of the studies included in this umbrella review also maintained this division, focusing narrowly on either physical design indicators or experiential dimensions such as familiarity and perceived safety.

By contrast, our synthesis—drawing on frameworks such as Gan et al.’s (2022) Planning and Design Principles for Dementia-Friendly Environments—illustrates how measurable NBE features (e.g., accessibility, navigability, sensory quality) can be aligned with experiential aspects (e.g., familiarity, safety, autonomy) to create supportive environments for PlwD and people with MCI. This framework offers a valuable means of integrating both quantitative and qualitative evidence within a unified design perspective.

Future studies should explicitly employ mixed-methods designs to operationalise this integration, for example, by combining spatial metrics with qualitative assessments of comfort, identity, or social meaning. Validated instruments such as the Lubben Social Network Scale or De Jong Gierveld Loneliness Scale could be integrated into future research to measure the influence of NBE on individual dimensions of social health [48, 49].

### NBE and cognitive/social health: mechanisms, conflicting findings, and research gaps

In line with the framework of pathways of health-promoting mechanisms of action (Frank et al. 2019), most NBE features indirectly affect cognitive and social health through behaviours.

These four health-promoting mechanisms are: (1) promotion of physical activity, (2) promotion of social interaction, (3) reduction of stress, and (4) enhancing self-determination and independence [24].

Green and blue infrastructure consistently emerged as key NBE components supporting cognitive and social health. Conversely, high population and building density, while often considered in quantitative studies, showed mixed associations—some studies link it to overcrowding and dementia risk, whereas others suggest that greater urban density may enhance cognitive stimulation through better access to services and social opportunities.

These conflicting findings suggest that the impact of density and related urban characteristics is highly context-dependent. Beneficial aspects, such as increased accessibility and opportunities for social engagement, may coexist with adverse effects, including crowding and sensory overload. This highlights the need for multidimensional measures that capture both positive and negative environmental attributes.

Similarly, the evidence for other NBE criteria, such as land-use mix, accessibility to local services, and transportation infrastructure, remains inconsistent. While some studies report that these features promote social interaction and independence, others find little or no association with cognitive outcomes. These discrepancies may stem from differences in study design, measurement, and population characteristics, underscoring the importance of contextual interpretation.

A further research gap lies in the contrasting focus of quantitative and qualitative studies. Quantitative research tends to rely on objective spatial indicators, providing measurable but often decontextualised associations. Qualitative studies, on the other hand, reveal the nuanced experiences of PlwD and their caregivers, including challenges of orientation, wayfinding, and perceptions of safety, but lack generalisability. Integrating these approaches would allow for a more comprehensive understanding of how environmental features function in everyday life.

### Under-researched indicators

The analysis revealed that certain NBE indicators remain underexplored, especially wayfinding infrastructure and neighbourhood physical disorder. Although frequently mentioned in qualitative studies as important for daily navigation and perceived safety, they have rarely been investigated quantitatively. This imbalance limits understanding of how such features contribute to or hinder cognitive and social health.

In particular, physical disorder—such as neglected public spaces, poor maintenance, or vandalism—can influence how people perceive and use their environment, potentially affecting both emotional well-being and social connectedness. Similarly, wayfinding elements such as signage, landmarks, and street layout are crucial for maintaining autonomy in older age but remain insufficiently assessed. These aspects should be prioritised in future studies to achieve a more complete understanding of supportive neighbourhood design. Future studies should prioritise these indicators and examine how they interact with behavioural and psychosocial pathways, for instance, by assessing whether improved wayfinding reduces stress or enhances independence among PlwD and people living with MCI.

The findings align with Ward et al. (2017) and Wu et al. (2020) in showing that NBE features like care services, transportation, shops, and recreational areas can support social participation and reduce dementia progression risk. However, this review also highlights underexplored aspects and calls for more standardised quantitative research to clarify their impact on cognitive and social health [6, 19]. Taken together, the evidence demonstrates that while some NBE components—particularly green and blue spaces—are consistently associated with positive outcomes, others remain insufficiently studied or context-dependent, revealing important directions for future research.

### Strengths and limitations

A significant strength of this review is its comprehensive summary and assessment of evidence on NBE features related to cognitive functioning and dementia at the ‘tertiary level’ (umbrella review) [50]. The results were organised using the ecological model of cognitive health according to Cerin (2019), supplemented by two further inductive categories, facilitating a broader and more conceptually grounded overview. These inductive categories—wayfinding infrastructure, and neighbourhood physical disorder—introduce dimensions that extend existing ecological models and point toward future refinements.

However, umbrella reviews have methodological weaknesses in summarising evidence from primary studies. This review’s narrative synthesis limits its methodological quality [51]. Fifteen primary studies were included across several reviews, implying some knowledge has been aggregated multiple times. The review by Zhao et al. (2022) could not be checked for duplicates at the primary study level due to an inaccessible appendix. Despite these limitations, the synthesis provides a valuable higher-level perspective on common methodological and conceptual gaps across the evidence base. Appendix A includes a table (Table 1) listing the multiple included studies in the reviews.

## Conclusions

In Cerin’s (2019) simplified ecological model of cognitive health, the neighbourhood-built environment influences cognitive health through multiple mediating factors [27]. This umbrella review supports and extends that framework by showing how NBE features contribute to cognitive health through physical activity, social interaction, mental engagement, and perceived safety. It further highlights under-researched yet conceptually important indicators, such as wayfinding infrastructure and neighbourhood physical disorder, that may affect autonomy, emotional well-being, and social participation.

According to the model of Frank et al. (2019), the NBE affects social and cognitive health through specific health-promoting mechanisms. Most observed associations between NBE factors and cognitive health are explained by health behavioural mediators such as physical activity, cognitive stimulation, and social connectedness, reinforcing the role of the built environment as a modifiable determinant of health. Together, these findings underline that an integrated understanding of objective and subjective NBE features is essential for promoting dementia-friendly urban design.

### Recommendations

Based on the reviewed evidence, several practical recommendations can be proposed:

1. Integrate health-promoting features into urban design: Urban planning should prioritise walkable, safe, and socially engaging neighbourhoods with accessible services and green or blue spaces that encourage physical activity and social interaction. These elements support independence and cognitive health for PlwD and people with MCI.
2. Advance dementia-friendly interventions: The complex topics identified in this review—particularly wayfinding infrastructure and neighbourhood physical disorder—require further investigation through large-scale, longitudinal primary studies. Insights from such research can inform evidence-based environmental interventions that promote autonomy and quality of life for people with MCI and PlwD.
3. Adopt participatory and mixed-method approaches: Future studies and tool development should actively involve PlwD and people with MCI to ensure that interventions and measurement instruments reflect real-world experiences and challenges [52]. The integration of quantitative spatial metrics with qualitative feedback has the potential to facilitate a more comprehensive understanding of the methodological divide that currently exists.
4. Encourage interdisciplinary collaboration: Urban planners, health professionals, and researchers should work jointly to translate evidence into practical strategies, ensuring that built environments foster cognitive, social, and overall well-being for PlwD and people with MCI.

Ultimately, neighbourhood-built environment factors are central to enabling older adults, and especially those with cognitive impairment, to live independently, safely, and meaningfully. Continued interdisciplinary research will be critical to turning this evidence into actionable urban design and policy frameworks.

## Supplementary Information

Supplementary Material 1.

## Data Availability

No datasets were generated or analysed during the current study.

## Appendix

### Search string MEDLINE (Pubmed)

“TITLE dementia OR alzheimer*” OR “vascular dementia” OR “frontotemporal dementia” OR “lewy body dementia” OR “Mild Cognitive Impairment” OR “cognitive impairment*” OR “cognitive decline” OR “cognitive ageing”) OR ABS (dementia OR alzheimer* OR “vascular dementia” OR “frontotemporal dementia” OR “lewy body dementia” OR “Mild Cognitive Impairment” OR “cognitive impairment*” OR “cognitive decline” OR “cognitive ageing”) AND TITLE (“built environment” OR “neighbourhood environment” OR “physical environment” OR “outdoor environment” OR “neighbourhood characteristics” OR “neighbourhood space*” OR “urban design” OR “environment design” OR “dementia-friendly” OR “land use” OR walkability OR “transport infrastructure” OR “pedestrian environment” OR “blue space*” OR “green space*”) OR ABS (“built environment” OR “neighbourhood environment” OR “physical environment” OR “outdoor environment” OR “neighbourhood characteristics” OR “neighbourhood space*” OR “urban design” OR “environment design” OR “dementia-friendly” OR “land use” OR walkability OR “transport infrastructure” OR “pedestrian environment” OR “blue space*” OR “green space*”).

## Abbreviations

AD: Alzheimer’s Disease
MCI: Mild cognitive impairment
MMSE: Mini-Mental State Examination
NBE: Neighbourhood-built environment
PlwD: People living with Dementia
