# Supplementary material for: Neighbourhood-built environment and cognitive or social health in older adults with mild cognitive impairment or dementia: an umbrella review
Source: BMC Geriatr. 2025 Nov 15;25:907. doi: 10.1186/s12877-025-06693-z (PMC12619174; doi:10.1186/s12877-025-06693-z)
Supplement: Supplementary file 1 — Supplementary Material 1. [file 12877_2025_6693_MOESM1_ESM.docx]

**Appendix A**

**Search string MEDLINE (Pubmed)**

“TITLE dementia  OR  alzheimer*”  OR  "vascular dementia"  OR  "frontotemporal dementia"  OR  "lewy body dementia"  OR  "Mild Cognitive Impairment"  OR  "cognitive impairment*"  OR  "cognitive decline"  OR  "cognitive ageing" )  OR  ABS ( dementia  OR  alzheimer*  OR  "vascular dementia"  OR  "frontotemporal dementia"  OR  "lewy body dementia"  OR  "Mild Cognitive Impairment"  OR  "cognitive impairment*"  OR  "cognitive decline"  OR  "cognitive ageing" )  AND  TITLE ( "built environment"  OR  "neighbourhood environment"  OR  "physical environment"  OR  "outdoor environment"  OR  "neighbourhood characteristics"  OR  "neighbourhood space*"  OR  "urban design"  OR  "environment design"  OR  "dementia-friendly"  OR  "land use"  OR  walkability  OR  "transport infrastructure"  OR  "pedestrian environment"  OR  "blue space*"  OR  "green space*" )  OR  ABS ( "built environment"  OR  "neighbourhood environment"  OR  "physical environment"  OR  "outdoor environment"  OR  "neighbourhood characteristics"  OR  "neighbourhood space*"  OR  "urban design"  OR  "environment design"  OR  "dementia-friendly"  OR  "land use"  OR  walkability  OR  "transport infrastructure"  OR  "pedestrian environment"  OR  "blue space*"  OR  "green space*" )

**Table 1** Inclusion and exclusion criteria of literature search

| Inclusion criteria | Exclusion criteria | Rayyan reason for exclusion |
| --- | --- | --- |
| **Reviews with a systematic methodical approach (Meta-analysis or narrative synthesis reviews; of both, quantitative or qualitative primary studies)** | Review or concept papers without systematic methodical approach, i.e., regarding literature search, data extraction, synthesis  All primary study designs, comments, editorials | No systematic review |
| **Reviews of primary studies in which community-dwelling elderly persons with dementia, MCI or cognitive impairments and/or their informal caregivers were interviewed on the influence of features of the built environment** | Reviews of primary studies focusing on built environment features of long-term care facilities, i.e., interior design elements of nursing homes, inpatient rehabilitation facilities, assisted living facilities (ALF), hospice, senior day care services, and long-term acute care hospitals as well as their surroundings | Wrong setting |
| **Reviews on primary studies focusing primarily on elderly populations (adults aged ≥45 years)** | Studies that address earlier life phases with population primarily younger than 45 years | Wrong age group |
| **Reviews of primary studies addressing the influence of features of the built environment on elderly people with cognitive impairment and on persons living with dementia in one of three ways:**  **influence of built environment features on cognitive functioning**  **influence of built environment features on psycho-social outcomes, i.e., quality of life (QoL), well-being or social participation, behavioural and psychological symptoms in dementia**  **subjective experience of respondents about features of the built environment** | Studies that address none of these thematic focuses | Wrong topic/outcome |
| **no restrictions regarding publication year** | - | - |
| **peer-reviewed journal publications** | - | - |
| **English and German language** | The study was published in a language not accessible to the reviewers | Wrong language |
|  | The staff performing the systematic review were unable to obtain the full text of the article. | Article not available |

**Table 2** Duplicates of the systematic literature research

| Primary study |  |  | Reviews |
| --- | --- | --- | --- |
| Author | **Year** | **Title** | **Authors** |
| Blackman et al. | 2007 | Outdoor environments for people with dementia: An exploratory study using virtual reality. | Keady, Gan |
| Blackstock et al. | 2006 | Living with dementia in rural and remote Scotland: Diverse experiences of people with dementia and their carers. | Keady, Gan |
| Brorsson et al. | 2011 | Accessibility in public space as perceived by people with Alzheimer’s disease. | Keady, Gan |
| Brown et al. | 2018 | Health disparities in the relationship of neighbourhood greenness to mental health outcomes. | Chen, Besser, Besser et al. |
| Cherrie et al. | 2018/2019 | Green space and cognitive aging: a retrospective life course analysis in the Lothian birth cohort (2018, Besser, Kim, Chen) Association between the activity space exposure to Parks in childhood and adolescence and cognitive aging in later life (2019, Besser, Chen) | Besser, Chen, Kim |
| Clarke et al. | 2012 | Cognitive function in the community setting: the neighbourhood as a source of "cognitive reserve. | Besser et al., Chen, de Keijzer |
| Dadvand | 2015 | Green spaces and cognitive development in primary schoolchildren. | de Keijzer, Besser |
| Duggan et al. | 2008 | The impact of early dementia on outdoor life: a "shrinking world"? | Keady, Sturge, Gan |
| Mitchell et al. | 2004/2006 | Dementia-friendly cities: Designing intelligible neighbourhoods for life. (2004, Gan, Keady) Neighbourhoods for life: Designing dementia-friendly outdoor environments (2006, Gan, Keady) | Gan, Keady |
| Olsson et al. | 2013 | Persons with early-stage dementia reflect on being outdoors: a repeated interview study | Sturge, Mmako |
| Smith et al. | 2016 | Developing a dementia-friendly Christchurch: Perspectives of people with dementia. | Sturge, Gan |
| Ward et al. | 2018 | The lived neighbourhood: understanding how people with dementia engage with their local environment. | Gan, Mmako, Sturge |
| Wu et al. | 2015/2017 | The built environment and cognitive disorders: Results from the cognitive function and aging study. (Gan, Besser 2017) The association between community environment and cognitive function: a systematic review. (de Keijzer, Chen, Besser et al. 2015) Community environment, cognitive impairment and dementia in later life: results from the Cognitive Function and Ageing Study (Besser et al., Besser 2015) | Chen, de Keijzer, Gan, Besser, Besser et al. |
| Yuchi et al. | 2020 | Road proximity, air pollution, noise, green space, and neurologic disease incidence: a population-based cohort study. | Chen, Besser |
| Zhu et al. | 2020 | APOE epsilon 4 modifies the effect of residential greenness on cognitive function among older adults: a longitudinal analysis in China. | Chen, Besser |
